# CF2 transcription factor is involved in the regulation of Mef2 RNA levels, nuclei number and muscle fiber size

**DOI:** 10.1371/journal.pone.0179194

**Published:** 2017-06-15

**Authors:** Juan J. Arredondo, Jorge Vivar, Sara Laine-Menéndez, Leticia Martínez-Morentin, Margarita Cervera

**Affiliations:** Departamento de Bioquímica, Instituto de Investigaciones Biomédicas “Alberto Sols” UAM-CSIC and Centro de Investigación Biomédica en Red (CIBERER), c/ Arzobispo Morcillo 4, Facultad de Medicina, Universidad Autónoma de Madrid, Madrid, Spain; University of Minnesota Medical Center, UNITED STATES

## Abstract

CF2 and Mef2 influence a variety of developmental muscle processes at distinct stages of development. Nevertheless, the exact nature of the CF2-Mef2 relationship and its effects on muscle building remain yet to be resolved. Here, we explored the regulatory role of CF2 in the *Drosophila* embryo muscle formation. To address this question and not having proper null CF2 mutants we exploited loss or gain of function strategies to study the contribution of CF2 to Mef2 transcription regulation and to muscle formation. Our data point to CF2 as a factor involved in the regulation of muscle final size and/or the number of nuclei present in each muscle. This function is independent of its role as a Mef2 collaborative factor in the transcriptional regulation of muscle-structural genes. Although Mef2 expression patterns do not change, reductions or increases in parallel in CF2 and Mef2 transcript abundance were observed in interfered and overexpressed CF2 embryos. Since CF2 expression variations yield altered Mef2 expression levels but with correct spatio-temporal Mef2 expression patterns, it can be concluded that only the mechanism controlling expression levels is de-regulated. Here, it is proposed that CF2 regulates Mef2 expression through a Feedforward Loop circuit.

## Introduction

The formation of skeletal muscle during embryogenesis involves the commitment of mesodermal progenitors to the myogenic lineage and their fusion to form fibers followed by the expression of muscle structural genes [1–4]. In the *Drosophila* embryo, each muscle is constituted by a single fiber. They are physiologically identical, but have unique morphological characteristics, as size, number of fusions, shape, spatial orientation and attachment sites to the body wall [3–6].

In the last steps of *Drosophila* muscle development the highly regulated sarcomeric protein expression and sarcomere assembly are crucial to ensure proper thick and thin filament stoichiometry, so the adequate forces will be generated by each muscle [3,7]. However, even though all events should be very tightly controlled, little is known about the mechanisms that sense and adjust filament ratios. One point of control is the transcription of muscle structural genes, and the actions of various transcription factors, particularly at embryonic stages, have been characterized [4,8,9]. The MADS box protein Mef2 is a major player in muscle differentiation. In a *Mef2* mutant background, specification of muscle precursors proceeds normally but multinucleate myotubes are lacking [10–13]. Mef2 binding to their regulatory sequences is essential for the expression of structural genes such as *mhc*, *malc*, *mlc2 pm/mpm*, *Tn I* and *TnT*, *Tp1* or *act57B* in embryos and adult muscle tissues [13–18]. Mef2 expression starts during mid gastrulation, and after that, muscle specification occurs [11,19]. Mef2 expression is maintained throughout muscle specification and differentiation in every muscle cell [20,21]. Shortly after the identification of Mef2, it was also clear that activation of muscle-structural genes and muscle formation required not only different levels of Mef2 but a large amount of tissue specific transcriptional cofactors [9,22].

The *Drosophila Chorion factor 2*, a zinc finger transcription factor, was first identified through its repressor role in dorso-ventral patterning during oogenesis [23,24][24]. CF2 protein was the first *Mef2* collaborating factor characterized during embryogenesis [25]. Around mid-stage 12 (8–9 h AEL), after induction of *Mef2* expression, CF2 is detectable in both visceral and somatic mesoderm with a pattern similar to Mef2 and it is expressed in the nuclei of all three-muscle types [26]. In *Mef2* null mutants, muscle precursors are specified but no myoblast fusion and no *CF2* mRNA are detected, demonstrating that CF2 transcription is dependent, direct or indirectly, on Mef2 [26]. In the *Drosophila* embryo, Mef2 and CF2 synergistically activate a number of structural muscle genes and loss of CF2 function results in the reduction of their expression levels. The combination of Mef2 and CF2 has a synergistic effect on *actin 57B*, *Tn I*, and *mhc* transcription in embryonic muscles [25,27], while there are clusters of Mef2 and CF2 binding sites upstream of *troponin T*, *tropomyosin 1* and *2*, and *paramyosin* promoters [28]. Previous work reported impaired flight and deregulation in two hypomorphic *CF2* mutants [27,28]. In adult flight muscles, as in embryonic muscles, CF2 may participate in the fine-tuning of structural gene expression to ensure proper stoichiometry of contractile proteins and filament balance maintenance, contributing to the regulation of the fiber final size [27,28].

In summary, CF2 has been known for a long time to be a muscle expressed transcription factor involved in the regulation of sarcomeric gene expression. Despite the datasets acquired to date, our knowledge about the role of CF2 in muscle development is far from complete and many important questions, beyond its role as Mef2 cofactor, remain unsolved. In this paper, we investigated the role of the Zn finger transcription factor CF2 in muscle development in the *Drosophila* embryo and its contribution to muscle differentiation. We used RNA interference and gain of function strategies to address these questions. We demonstrated that, in addition to its contribution to *Mef2* transcriptional regulation of sarcomeric genes, CF2 is involved in the control of the fiber final size and in the regulation of the number of nuclei present in each individual muscle. CF2 over-expression causes an increase in muscle size and in the number of nuclei per fiber while CF2 down-regulation causes a decline in muscle size and nuclei number. In contrast, no increase in nuclei number is observed when Mef2 transcription factor is over-expressed.

## Materials & methods

### Fly strains, crosses and genetics

*Drosophila melanogaster* strains were reared at 25° on standard culture medium. We used the Gal4/UAS system [29] for tissue-specific expression of transgenes *UAS-CF2RNAi*, *UAS*-*CF2* and *UAS-Mef2* [10,30]. *Mef2*-*Gal4* [13] and *twi-Gal4;twi-Gal4* lines [31] were used as drivers. All lines used in this study are described below.

The pUAST-CF2 construct was generated from a full-length cDNA fragment of *CF2* flanked by NcoI sites cloned into NcoI sites in pUAST vector [29] and used to generate the *UAS-CF2* fly line. UAS-CF2RNAi construct was generated in two steps. First, a genomic *CF2* fragment containing exons 2 and 3, as well as introns 2 and 3, was cloned into pGEMTeasy vector, as a NotI and SfiI fragment, making the CF2RNAi Direct construct. To generate the CF2RNAicDNA Invert construction, an inverted fragment of the *CF2* cDNA containing exons 2 and 3 and flanked by SfiI / XbaI sites was cloned into pGEMTeasy vector. Then, Not/SfiI and SfiI/XbaI fragments from both plasmids were cloned into pUAST vector, generating the final pUAST- CF2RNAi vector.

Several independent homozygotes *UAS-CF2* and *UAS-CF2 RNAi* lines were generated by P-element mediated transformation using standard procedures [32] and *yw* as host. In the analysis, all of them showed the same phenotype. Df2 γ^27^ deficiency [24–26], *UAS-Mef2* [10,30] *Mef2*-*Gal4* [13] and *twi-Gal4*; *twi-Gal4* lines [31,33] were previously described. All lines used in the study were balanced with GFP or LacZ marked chromosomes (Tm3Ser*Act5C*-GFP and Cyo*WgLacZ*) for embryo genotype selection (Table 1).

**Table 1:** List of Drosophila lines used in this study.

Line	Genotype
Mef2>CF2i	UAS- CF2RNAi; Mef2-Gal4/TM3SerAct5C-GFP
Mef2>CF2	UAS-CF2; Mef2-Gal4 / TM3Ser Act5C-GFP
twi>CF2	twi-Gal4; twi-Gal4; UAS-CF2 / Tm3SerAct5C-GFP
twi>CF2i	twi-Gal4; twi-Gal4; UAS-CF2RNAi / Tm3SerAct5C-GFP
Mef2>Mef2	UAS-Mef2; Mef2-Gal4 / TM3SerAct5C-GFP
Df2©^27^	Df2©^27^ / CyoWgLacZ

### Quantitative RT-PCR

**Fig 1:**
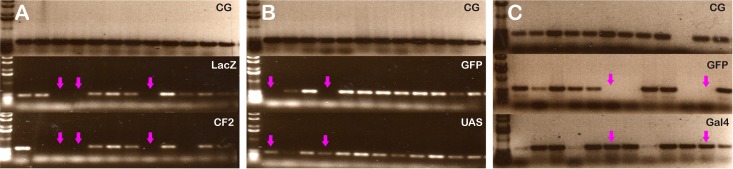
Genotype identification of an individual embryo. The presence or absence of the balancer chromosome was assayed by conventional PCR in individual embryos carrying the Dfγ^27^ deficiency, UAS-CF2 or Gal4 insertions. Homozygous embryos are indicated with arrows. For all three fly lines the upper part of the panels show a positive control PCR against a single copy CG9650 unrelated gene. (A). Dfγ^27^ embryos. The middle part of the panel shows the result obtained when the balancer marker gene LacZ was amplified. The lower part of the panel presents the results for the *CF2* gene. Only those embryos showing no amplification of both genes were considered homozygous for the Dfγ^27^ deficiency. (B). UAS-CF2. The middle part of the panel shows the results obtained when the balancer marker gene GFP was amplified. The lower part of the panel shows the result for UAS sequence from the UAS-CF2 insertion. Only those embryos showing no GFP amplification and the presence of the UAS region were considered to carry the UAS-CF2 insertion in both chromosomes. (C). *Gal4* driver lines. The middle part of the panel shows the results obtained when the balancer marker gene GFP was amplified. The lower part of the panel shows the result for *Gal4* sequence in the driver. Only those embryos showing no GFP amplification and the presence of the *Gal4* region were considered to carry the two copies of the driver, one in each chromosome.

Individual embryos from 12 to 14 hours of development were genotyped according to the presence or absence of the balancer chromosome marker gene, *GFP* or *LacZ*, detected by conventional PCR as described previously [34]. Embryos displaying the absence of the balancer chromosome marker gene, LacZ plus absence of CF2 (Fig 1A) or GFP plus presence of UAS (Fig 1B) or Gal4 (Fig 1C) were selected (see magenta arrows in Fig 1A–1C). Note that in *Df2*©^*27*^ homozygous embryos CF2 gene is absent. Next, 5 homogenates of individual genotyped embryos of each desired genotype were pulled together and total RNA was isolated using an RNeasy protect mini kit (Qiagen). First-strand cDNA was primed with poly dT and SuperScript III (Invitrogen) according to manufacturer's instructions. Quantitative PCR was carried out with TAQMan probes (Invitrogen) according to manufacturer's standard conditions in an ABI Prism 7900HT instrument (Applied Biosystems). rRNA 18S was used as reference. Measurements were performed in triplicates and mean results were plotted as 2^-ΔΔCt^ relative to the wild type level [35].

### Immunohistochemistry

Immunohistochemistry analyses were performed as previously [36,37]. Polyclonal rabbit CF2 antibody was produced in our laboratory. A His-CF2 fusion protein was produced from the pRSETB-CF2 construct and purified in HisTrapHP columns (GE, HealthCare). After standard immunization protocol, CF2 serums were affinity purified using Affigel 10–15 (BioRad). The following antibodies were used at indicated dilutions: polyclonal rabbit anti-CF2 (1/500), anti-TnT (1/1000), anti-Mef2 (1:1000, kindly provided by G. Melkhiany from Bodmer's lab), anti MHC (1/1000 kindly provided by Bernstein's lab), mouse monoclonal anti-Eve (2B8, 1/20, Developmental Studies Hybridoma Bank), anti GFP (1/1000, Roche) and anti β-galactosidase (1/2000, Promega). Goat anti-rabbit or anti-mouse Alexa Fluor 647, 546 and/or 488 (1:1000) from Molecular Probes, were used as secondary antibodies in different combinations. Experiments were carried out at least 6 times to rule out that observed differences could be due to technical issues. All staining were performed following exactly the same protocol for both staining and acquisition. The *yw* control was carried along in every experiment. Dozens of embryos were assayed in each experiment and they always showed mendelian proportion according to the parental genotypes.

Samples were analyzed in Leica TCS-SP2 and Leica TCS-SP5 Confocal systems. All presented pictures were collected using the same settings and images were equally processed. All presented pictures correspond to maximum projections collected with maximum intensity.

### Statistics

Data are expressed as mean ± SEM obtained from, at least, three separate, independent experiments carried out in different days and with different preparations. The statistical analyses *p-*values were generated using Student's *t-*test (unpaired, 2-tailed), using the SPSS 17.0 for statistical program (SPSS Inc.); p*-*value < 0.05 was considered significant.

## Results

### CF2 interference or overexpression generates muscle phenotypes in *Drosophila* embryos

*CF2* expression onset has been described to initiate just after stage 11, 7.5 hours after egg laying (AEL), coinciding with skeletal myoblast fusion [26,38]. To more precisely delineate *CF2* function in muscle development, and using Gal4-UAS system, we carried out two complementary approaches. On the one hand we interfered CF2 expression and on the other hand we performed gain of function experiments, both followed by phenotype analysis. Since CF2 expression pattern fully overlaps with that of Mef2, we selected *Mef2-Gal4* and *twi-Gal4* drivers for both approaches [26]. First, we generated stable fly lines for the two drivers: *Mef2-Gal4; UAS-CF2RNAi* and *twi-Gal4; UAS-CF2RNAi*, both carrying a *TM3SerAct5c-GFP* balancer that allowed us to identify homozygous embryos, those not expressing GFP. In homozygous animals, carrying two copies of *UAS-CF2RNAi*, *CF2* knockdown (KD) driven by two copies of *Mef2* driver resulted in embryonic lethality while *CF2* KD driven by two copies of *twi* driver caused lethality at 3^rd^ larval stage. Since we interpreted these results as the consequence of *twi* driver being weaker and hence achieving lower interference levels, we decided to introduce another driver copy. Homozygous *twi-Gal4; twi-Gal4*, *UAS-CF2RNAi* embryos turned out to be early embryonic lethal (not shown). Unless otherwise mentioned, all interfered embryos shown in this work are homozygous for both drivers and for UAS-CF2RNAi insertions, from now on they will be referred as Mef2>CF2i or twi>CF2i. We also analyzed the *Df2©*^*27*^ line that carries a 25kb deletion covering the entire CF2 locus plus 20 different loci more and it is homozygous larval lethal [24,26].

In order to perform gain of function experiments, we generated fly lines heterozygous for *Mef2-Gal4* and *twi-Gal4* drivers and homozygous for the UAS-CF2 insertion. They will be referred to as Mef2>CF2 or twi>CF2.

**Fig 2:**
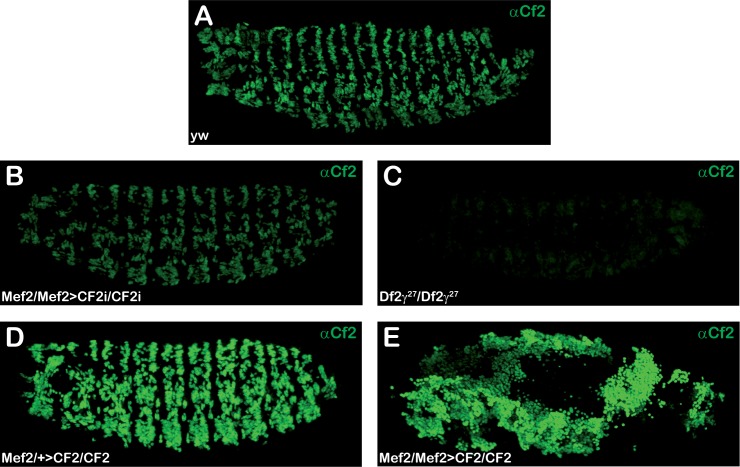
Different CF2 protein levels in CF2 interfered and overexpressed embryos. Lateral views of stage 17 embryos stained with anti-CF2 antibody are shown. (A). *yw* embryo. (B). Mef2>CF2i homozygous embryo. (C). *Df2γ*^*27*^ homozygous embryo. (D). Mef2>CF2 heterozygous embryo, carries only one driver copy. (E). Mef2>CF2 homozygous embryo, carries two driver copies. Anterior to the left and posterior to the right. In panel D, + stands for *TM3Ser Act5C-GFP*. Pictures were collected using the same settings and images were equally processed. They correspond to maximum projections collected with maximum intensity.

**Fig 3:**
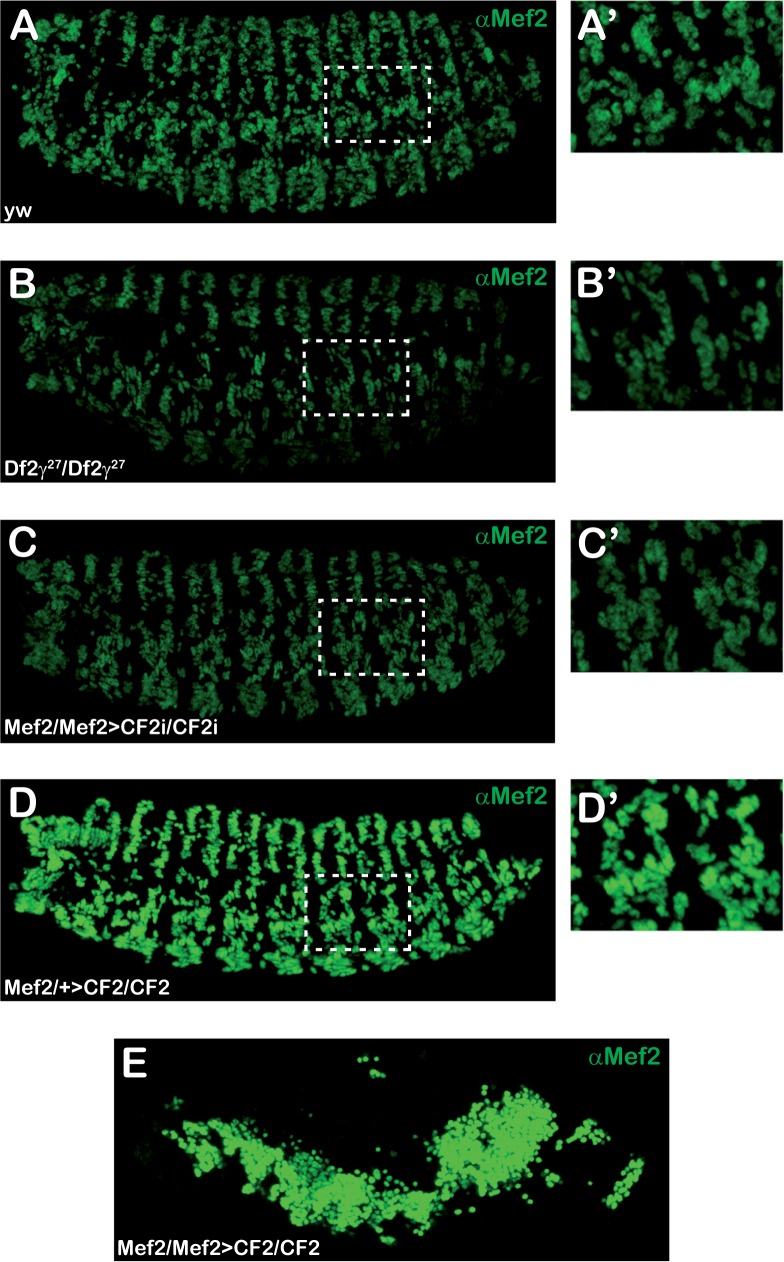
Mef2 expression in CF2 interfered and overexpressed embryos. Lateral views of stage 17 embryos stained with anti-Mef2 antibody are shown. (A). *yw* embryo. (B). *Df2γ*^*27*^homozygous embryo. (C). Mef2>CF2i homozygous embryo. (D). Mef2>CF2 heterozygous embryo. (E). Mef2>CF2 homozygous embryo carrying two driver copies (A’-D’) Amplification detail from de dotted region shown in A-D panels. Anterior to the left and posterior to the right (A-E). In panel D, + stands for *TM3Ser Act5C-GFP*. Pictures were collected using the same settings and images were equally processed. They correspond to maximum projections collected with maximum intensity.

CF2 KD and overexpression (OE) at early stage 17 (17–18 hours AEL) were examined by CF2 immuno-staining. As shown in Fig 2, although no obvious morphological defects can be detected, CF2 expression in Mef2>CF2i embryos was clearly reduced as compared with *yw* control embryos. Moreover, as expected, homozygous *Df2©*^*27*^ embryos showed no CF2 expression (compare Fig 2A–2C). On the contrary, heterozygous Mef2>CF2 embryos showed a strong increase in CF2 expression (Fig 2D), while double homozygous embryos for both insertions, UAS-CF2 and Mef2-Gal4, were early embryonic lethal (Fig 2E) and showed a very strong disorganization. Accumulations of CF2 expressing cells with no obvious organization were present in late embryos. These cells also displayed a strong Mef2 expression, suggesting that they have a mesodermal origin (see below, Fig 3). Same results were obtained in twi>CF2i and twi>CF2 (see S1 Fig).

### CF2 regulates Mef2 expression during embryo skeletal myogenesis

It has been previously reported that *Mef2* expression is not affected in *Df2©*^*27*^ homozygous embryos [26]. In order to confirm those results and to more precisely define muscle patterns in embryos lacking *CF2*, we decided to analyze *Mef2* expression in Mef2>CF2i and homozygous *Df2©*^*27*^ embryos. Surprisingly, as shown in Fig 3, Mef2 embryo immunostaining revealed an important reduction in *Mef2* expression in both, Mef2>CF2i and *Df2©*^*27*^ embryos, when contrasted to wild type (Fig 3A–3C). Since the reduction is observed in both CF2 KD and *Df2©*^*27*^ embryos, we can conclude that the Mef2 diminution observed in the later was in fact due to the lack of CF2 and not that of any of the other genes deleted by the deficiency (Fig 3B).

On the contrary and corroborating these results, Mef2>CF2 embryos, displaying high levels of CF2, showed a clear increase in Mef2 expression as compared to wild type (Fig 3D). These results supported that Mef2 levels are undoubtedly related to those of CF2, suggesting the existence of a regulatory feedback loop among these transcription factors.

**Fig 4:**
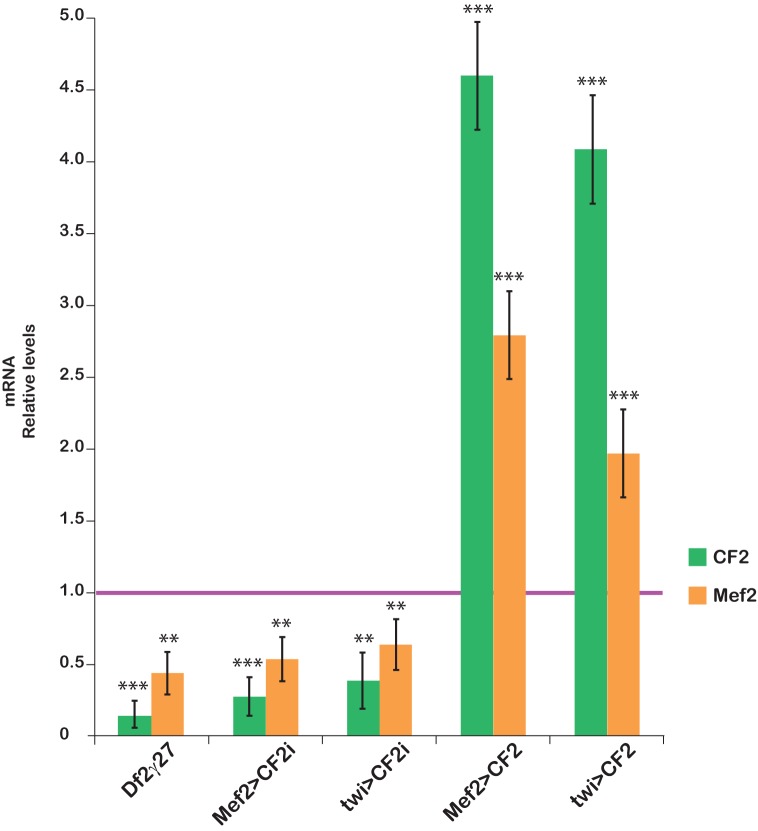
CF2 and Mef2 RNAs correlated in the interfered and overexpressed CF2 embryos using real-time quantitative PCR. mRNA relative levels of CF2 (green) and Mef2 (orange) in the deficiency (Df2γ^27^), the interference (Mef2>CF2i and twi>CF2i) and the over-expression (Mef2>CF2 and twi>CF2) fly lines relative to CF2 expression in the *yw* control line (magenta line, value = 1). Measurements were carried out in triplicate. 18S was used for standardization. Mean results were plotted as 2^-ΔΔCt^ (n = 4). Statistical significance of expression variation as compared to yw control: *p<0.05; **p<0.005; ***p<0.001.

It is well known that CF2 and Mef2 collaborate in the activation of several structural muscle genes in *Drosophila* [25]. It is therefore not difficult to imagine that both transcription factors interact with each other to regulate their own transcription, regardless of whether this interaction is direct or indirect. Nevertheless, the results described above are also compatible with a CF2 dependent stabilization of Mef2 protein. Bearing this in mind, to confirm and reinforce the idea of the existence of a regulatory feedback loop in which CF2 modulates Mef2 expression, we have investigated if the variation in Mef2 protein levels correlates with a variation in RNA levels. Thus, we determined by qPCR CF2 and Mef2 expressions levels in *Df2©*^*27*^, CF2 KD and CF2 OE embryos. Stage 15–16 embryos were individually genotyped in order to select embryos homozygous for *Df2©*^*27*^, twi>CF2i, Mef2>CF2i, twi>CF2, Mef2>CF2, or heterozygous for Mef2-Gal4 and homozygous for UAS-CF2 (see Material and Methods and Fig 1/data not shown). Embryos of the desired genotype were pulled together in groups of five and expression levels of both genes were analyzed by qRT-PCR. At least three independent pulls of each genotype were analyzed in triplicates. Results are shown in Fig 4. As expected, *CF2* expression levels in CF2 KD lines are strongly reduced. When the interference is driven by Mef2-Gal4, *CF2* RNA levels are reduced to approximately 25% of that present in wild type, whereas in *twi* driven interference 40% of *CF2* RNA remains (Fig 4). As expected, homozygous *Df2©*^*27*^ embryos show no CF2 expression.

Interestingly, interfered embryos, as it happens in homozygous *Df2©*^*27*^, display a strong reduction in Mef2 expression level, between 50% and 65% of the one observed in the control. Accordingly, it is important to note that, although not statistically significant, there seem to be a downward trend in which Mef2 expression reduction is higher in *Df2©*^*27*^ homozygous embryos and lower when the interference is driven by *twi-*Gal4 (Fig 4). Moreover, CF2 overexpression induces Mef2 overexpression regardless of the driver used, *Mef2*-Gal4 or *twi*-Gal4 (Fig 4). As it happens with CF2 interference, levels of CF2 OE and the concomitant ones of Mef2 are higher in the Mef2>CF2 lines (Fig 4). These results undoubtedly show that CF2, in a direct or indirect manner, regulates Mef2 expression. Moreover, they could support the existence of a feedback loop between both factors, being the expression of each of them dependent on the other. Since Mef2 and CF2 expression initiate at the myoblast fusion stage, the cooperation of these two factors should affect the next steps of the myogenic program, as indeed happens to gene expression regulation of sarcomeric genes [16,25,27,28].

### CF2 is involved in skeletal muscle size and number of nuclei per fiber regulation

In the adult fly, two separate functions have been proposed for CF2. On the one hand, fine-tuning the expression of structural genes to ensure proper filament stoichiometry, and on the other hand monitoring and/or controlling the final myofibril size [27]. Therefore, we wondered if that would be the case in embryo muscles too. To define CF2 contribution to the regulation of the final fiber-size in the embryo we studied muscle shape and size in CF2 KD or OE embryos using myosin heavy chain and TnT immunostaining to visualize muscles in stage 16–17 embryos.

**Fig 5:**
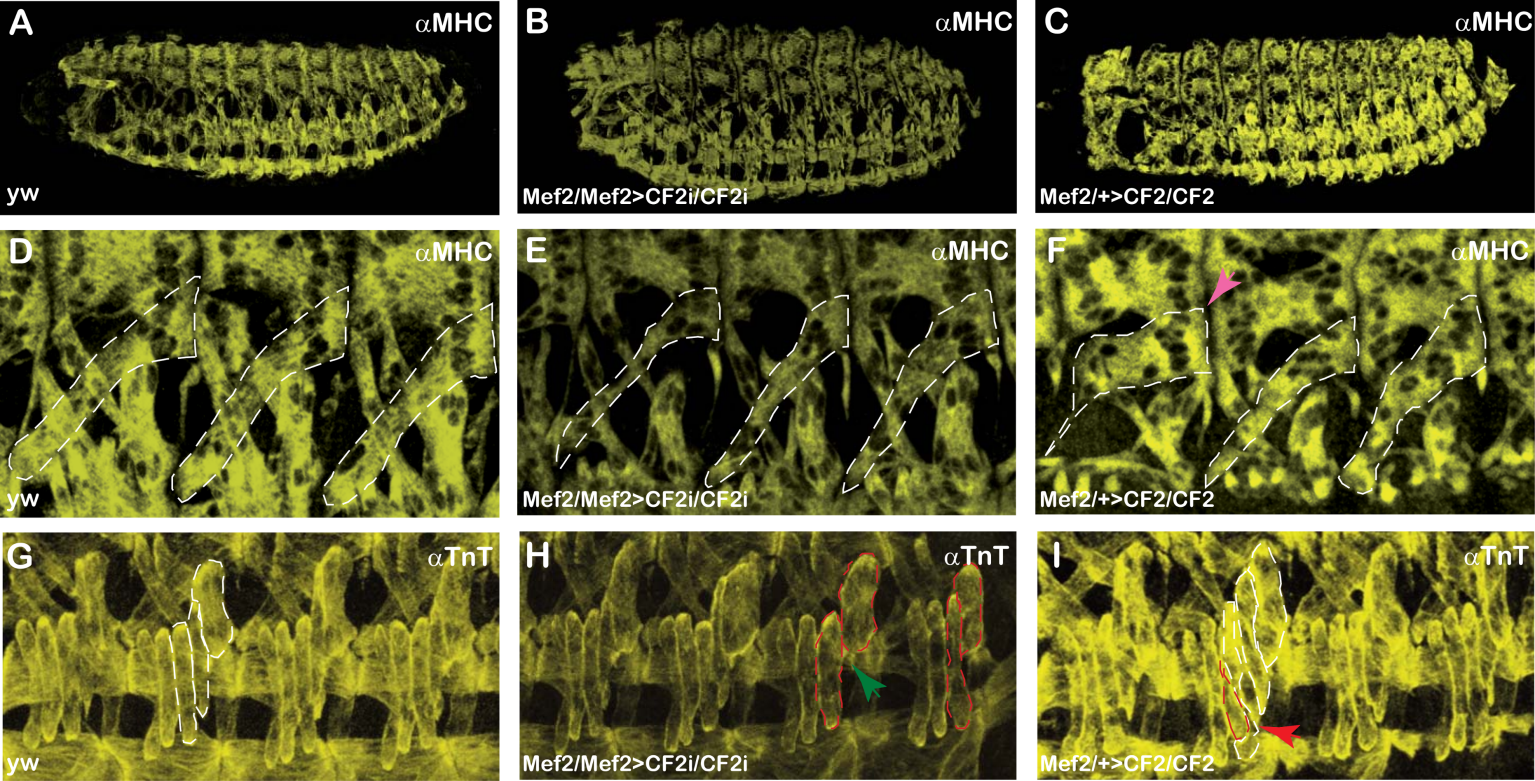
CF2 is involved in somatic muscle size. Anti-MHC (A—F) or anti-TnT (G—I) inmunostaining of stage 17 embryos. (A, D, G) *yw* embryos. (B, E, H) Mef2>CF2i homozygous embryos. (C, F, I) Mef2>CF2 heterozygous embryos, carrying one driver copy. (A—C) Whole embryos, lateral views, anterior to the left and posterior to the right. (D–F) Magnification presenting muscles from segments A2-A4 in the lateral region. DA3 muscle is outlined. (G—I) Magnification showing LT1-4 and DT1 muscles from segments A2-A4 in the lateral region. Pink arrow in panel F indicates alteration in morphology. LT3, LT4 and DT1 are indicated with white discontinued lines in panel G. The LT4 absence is indicated with a green arrow in panel H and the presence of a new muscle is indicated with red arrow and red discontinued lines in panel I. Pictures were collected using the same settings and images were equally processed. They correspond to maximum projections collected with maximum intensity.

As compared to wild type, Mef2>CF2i embryos presented lower MHC expression, little defects in the overall skeletal musculature and smaller muscles (compare Fig 5A and 5B and Table 2). A closer inspection of those embryos revealed that not only muscles were smaller, but also, in most cases, they had a smaller number of nuclei (compare outlined DA3 muscles in segments A2-A4 in Fig 5D and 5E and see below). Moreover, in some cases, fibers are absent (green arrow pointing absent LT4 muscle in Fig 5). It is important to notice that despite being smaller, muscle shape and anchoring points seems not to be altered (Fig 5H). This lower number of nuclei is also apparent when embryos are stained for Mef2 expression. As shown in Fig 3, homozygous *Df2©*^*27*^ display a general decrease in the number of nuclei positive for Mef2 expression (compare Fig 3A’, 3B’ and 3C’) and smaller size (see Table 2). As opposed to CF2 KD, in Mef2>CF2 embryos overexpressing CF2, fiber size is increased (Table 2), presenting a higher nuclei number and, most important, even though the fiber shape and anchoring points are normal (see pink arrow in Fig 5F), we observed the appearance of new fibers (red arrow in Fig 5I).

**Table 2:** LT1-4 muscle size in μm^2^ from control, homozygous Df2g27, CF2i and CF2 OE embryos. LT1 to LT4 muscle size is given as mean +/- standard deviation. Statistical significance:. * p < 0.05; ** p < 0.005.

	Control	Df2*©*27/Df2*©*27	Mef2>CF2i	Mef2>CF2
LT1	410.85 +/- 62.4	347.54 +/- 60.6*	351. 21+/- 44.1*	467.13 +/- 51.1*
LT2	435.36 +/- 56.4	367.61 +/- 60.6**	373.95 +/- 64.4*	501.26 +/- 56.4*
LT3	414.00 +/- 67.2	332.78 +/- 74.9**	342.93 +/- 51.6*	531.88 +/- 49.4*
LT4	266.91 +/- 59.4	205.15 +/- 39.9*	228.64 +/- 65.2*	330.26 +/- 50.9**

To more precisely define these variations in nuclei number per fiber, consequence of the changes in CF2 expression levels, we measured the number of nuclei present in one particular fiber. Dorsal Acute 1 muscles (DA1) nuclei can be easily identified as the only skeletal muscle nuclei expressing high levels of Eve and Mef2 transcription factors [39,40]. However, Eve is expressed also in pericardial cells situated in the vicinity of DA1 muscles. Since pericardial cells do no express Mef2, to distinguish DA1 and pericardial cells nuclei, we performed co-staining with Eve and Mef2 antibodies.

**Fig 6:**
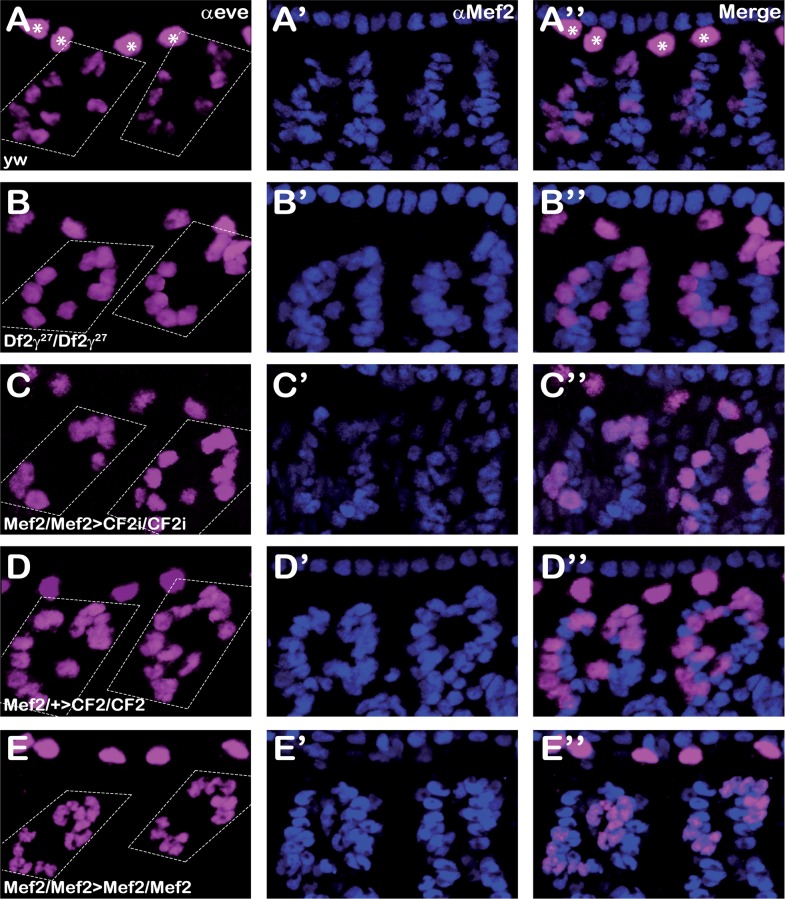
CF2 is involved in determining the number of nuclei in Dorsal Acute 1 muscle (DA1) from segments A3 and A4. Anti-eve (magenta) and Anti Mef2 (blue) staining of DA1 muscles from yw (A-A”), homozygous Df2γ27 (B-B”), homozygous Mef2>CF2i (C-C”), heterozygous Mef2>CF2 (D-D”) and homozygous Mef2>Mef2 stage 17 embryos. Left panels show *eve* stained nuclei, middle panels show Mef2 staining nuclei and right panels merge. Note that pericardial cells (*) do not express Mef2. It can be observed that eve expressing muscular nuclei number increase or decrease its number according to CF2 expression levels. In panels D, + stands for TM3Ser Act5C-GFP. Segments A3 and A4 are delimited by dotted lines. Pictures were collected using the same settings and images were equally processed. They correspond to maximum projections collected with maximum intensity.

Stage 16 embryos were double stained and the number of Eve/Mef2 positive nuclei in segments A3 and A4 counted. As shown in Fig 6, DA1 muscles from *Df2©*^*27*^ homozygous embryos, and therefore deficient in CF2, present a smaller number of nuclei, an average of 8 per fiber (see Table 3), as compared to control DA1 muscles, which contain 13 nuclei in average. In order to confirm that the lower number of nuclei was specifically due to the lack of CF2 and not to that of any of the other genes deleted by *Df2©*^*27*^ deficiency we analyzed the number of nuclei present in DA1 muscles from CF2 KD embryos. Regardless of the driver used, we observed a clear reduction in the number of Eve/Mef2 positive nuclei in CF2 KD embryos (Fig 6 & Table 3). According to the level of interference (see Fig 3B), the reduction observed in Mef>CF2i embryos was slightly stronger than that observed in twi>CF2i, with an average number of 10 and 11 nuclei respectively. When the same analysis was carried out in CF2 OE embryos, we observed the expected rise in the number of nuclei present in DA1 muscles (dotted squares in Fig 6 & Table 3). Again, in agreement to the level of over-expression, Mef2>CF2 embryos show a larger number of nuclei, an average of 19, than the twi>CF2 embryos (Table 3). Furthermore, a moderate Mef2 OE does not cause an increase in the number of nuclei present in DA1 muscles (Fig 6 & Table 3). Taken together, these results strongly suggest that CF2 is not only implied in muscle gene expression regulating the stoichiometry of contractile proteins [28], but regulating the number of nuclei in each fiber.

**Table 3:** Nuclei number in Dorsal Acute 1 muscle in interfered and overexpressed stage 17 embryos. Statistical significance: **p<0.005; ***p<0.001. ns–no significant.

Genotype	Number of nuclei (Mef2+ Eve+)
*yw*	13^±^ 1.6 (n = 20)
*Df2©*^*27*^	08 ^±^ 1.8 (n = 16) ***
*Mef>CF2i*	10 ^±^ 1.8 (n = 21) ***
*twi>CF2i*	11 ^±^ 2.0 (n = 18) **
*Mef>CF2*	19 ^±^ 1.2 (n = 25) ***
*twi>CF2*	16 ^±^ 2.1 (n = 20) **
*Mef>Mef2*	13 ^±^ 2.0 (n = 15) ns

## Discussion

Precursor muscle cells must activate and inactivate the expression of large cohorts of genes in a precise spatio-temporal manner to progress through muscle development. To achieve a molecular understanding of the regulatory networks controlling cellular decision-making it is essential to understand how inputs from different regulators are being integrated to define very precise patterns of gene expression [3,4]. The exact nature of the CF2 relationship with Mef2 and its effects on muscle building remain yet to be resolved. Here, we explored the regulatory role of CF2 in the embryonic *Drosophila* muscles and the contribution of CF2 to *Mef2* mRNA level regulation and muscle formation. To address this question we exploited tools of gain or loss of function. Our data point to CF2 as a factor involved in the regulation of muscle size as well as in the final number of nuclei present in each muscle. These functions are distinct of its role in the regulation of muscle-structural genes as Mef2 partner.

*Mef2* expression starts in the ventral furrow during gastrulation, stage 6, where is activated by *twist* [8,11,20]. Just after induction of Mef2 expression, around mid-stage 12 (8–9 AEL), the onset of CF2 expression occurs. Mef2 and CF2 patterns of expression are fully equivalent, with both proteins present in the nuclei of all three-muscle types [26]. By a cautious inspection of the Mef2 protein expression in Mef2>CF2i, twi>CF2i and *Df2©*^*27*^ embryos, we have demonstrated a clear decrease in Mef2 fluorescent signal, that is Mef2 protein abundance, in embryos where CF2 was expressed at lower levels or was absent (see Fig 3B’and 3C’ and compared with 3A’). Moreover, the opposite is also true and a proportional increase in fluorescent intensity was observed in embryos displaying higher levels of CF2 (Fig 3D’ and 3A’). Thus, the differences in CF2 amount are translated into comparative differences in Mef2 protein levels. Interestingly, regardless of CF2 expression being down or up regulated, Mef2 was expressed in every single myoblast just like in a wild type embryo (Fig 3, insert panels). Thus, Mef2 spatiotemporal expression pattern remains unaltered in response to CF2 fluctuations but its expression levels change. Along with those fluctuations there are two possible interpretations: *Mef2* transcriptional regulation is dependent on CF2 or Mef2 protein is stabilized by CF2. In fact, our qRT-PCR data reinforce the former one. Transcript analysis of individually genotyped CF2 KD and OE embryos confirmed that mRNA expression levels of both genes, Mef2 and CF2, vary in parallel. These results contrast with previous data in *Df2©*^*27*^ homozygous embryos that described no changes in Mef2 protein levels in absence of CF2 when comparing to those in wild type embryos [26]. They were based only in embryos double stained with CF2 and Mef2 polyclonal antibodies. Since the patterns of expression of Mef2 are exactly the same in wild type and in homozygous *Df2©*^*27*^ animals, one might think that CF2 does not influence in Mef2 expression levels. However, our embryo staining data together with qPCR analysis indicate beyond doubt that there is a relationship between CF2 and Mef2 expression levels and that CF2 is involved in Mef2 regulation. In fact, qPCR experiments on individually genotyped Df*©*^27^ homozygous embryos, the same deficiency previously used [26], show a clear decrease in Mef2 expression, ruling out any possible strain specific effects.

Cripps and collaborators proved the presence of a Mef2 dependent enhancer in the *Mef2* gene that directly and positively auto-regulates Mef2 transcription in muscle and allows sustained Mef2 expression [30]. In fact, this enhancer is required for muscle maintenance and growth so Mef2 auto-regulation and sarcomeric gene activations might be, through direct or indirect mechanisms, interconnected processes. Since our data revealed that CF2 was also required in these processes, we decided to search for potential binding CF2 sites in this enhancer. The presence of a putative CF2 binding site (GATATATAC) located 707 bp upstream of the Mef2 binding site described by Cripps was identified. According with Cripps data, when the CF2 binding-site containing region is deleted, enhancer´s activity falls significantly. Moreover, the -8543/-8079 deletion analysis made by Cripps and collaborators indicated complete absence of activity. The analyzed fragment contains the Mef2 binding site but not the CF2 binding site, which was deleted [30]. These results strongly support our hypothesis. Thus, Mef2 is essential, together with CF2, for muscle differentiation in *Drosophila* via direct activation of genes that have enhancers containing Mef2 binding sites as it is the case for muscle structural genes [13,14,17,18]. At that moment, Cripps and collaborators suggested that, in addition to Mef2, other unknown transcriptional factors were required for the autoregulatory mechanism. Later on, the synergistic effect of Mef2 and CF2 on *57B actin*, *Tn I*, and *mhc* genes in embryonic muscles together with the presence of clusters of conserved Mef2 and CF2 binding sites upstream of *troponin T*, *troponin I*, *tropomyosin 1* and *2*, MyHC, *paramyosin* and others were reported, hence validating the prominence of CF2 alongside Mef2 as a regulator of many structural muscle genes as well as in muscle sustainability [16,25,27,28].

**Fig 7:**
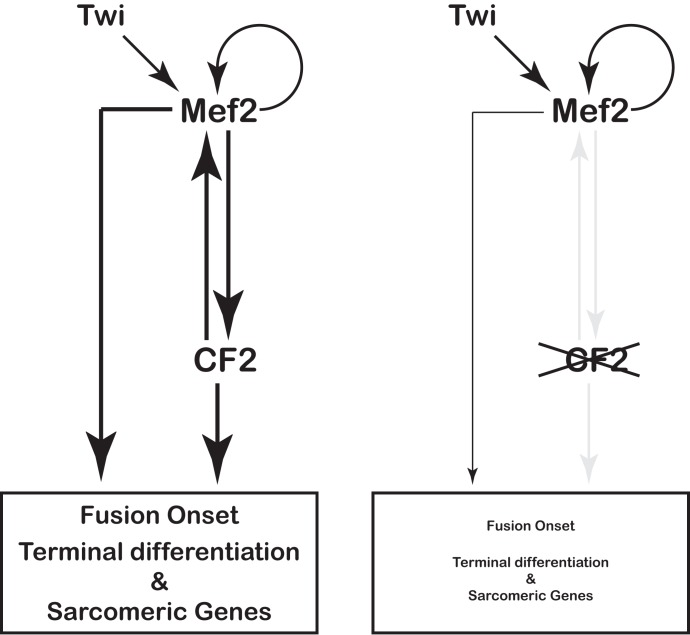
Proposed model for a mechanism of regulation in that CF2 regulates Mef2 expression through a Feedforward Loop (FFL) circuit. At stage 11, twi activates *Mef2* transcription which in turn activates its own transcription in a twi independent manner. At mid stage 12, Mef2 inducts CF2 transcription, which in turn increases Mef2 expression. Both transcription factors, Mef2 and CF2, cooperate to maintain high levels of Mef2 transcription and influence the fusion process. In the differentiated fiber, both factors collaborate in the regulation of sarcomeric genes expression (panel B). In the absence of CF2 (panel A), the feedback loop is lost and Mef2 expression is not increased by the action of CF2. Therefore, Mef2 expression relays only in the self-activation circuit, which renders low Mef2 expression levels with the concomitant impact on muscle fiber terminal differentiation, and in the regulation of sarcomeric genes expression.

Under the light of the available data from us and others, we propose a mechanism of regulation model in that CF2 regulates Mef2 expression through a Feedforward Loop (FFL) circuit (Fig 7) [41]. This model should be completely demonstrated in the future but fits well with our presented results. Thus, at stage 11, twi activates *Mef2* transcription, which in turn activates its own transcription in a twi independent manner. At mid stage 12, Mef2 induces activation of CF2 transcription. Both transcription factors, Mef2 and CF2, cooperate to maintain high levels of Mef2 transcription (Fig 7A). So, according to the proposed model, we speculate that in the absence of CF2 there is no FFL and therefore no stabilization of high Mef2 transcription levels whose transcription is kept at low levels through self-activation (Fig 7B). Moreover, modulation of Mef2 transcription levels would be CF2 concentration dependent. Hence, a fall in CF2 concentration would result in a concomitant decrease in Mef2 mRNA levels while a rise in CF2 concentration would have the opposite effect. Thus, according to the proposed mechanism, during the embryonic stages of muscle development, acting through enhancers containing clusters of Mef2 and CF2 binding sites, both factors should directly and positively regulate transcription of sarcomeric genes (Fig 7).

In *Drosophila*, developing myofibers are multinucleated syncytia that engage similar cellular mechanisms to become functional muscles [3–6]. Each muscle is constituted by a single myofiber and expresses a unique gene combination that provides them with specific features as size, shape, and function. Once the precursor muscle cells have been specified (by embryonic stages 11–12), cell–cell fusion occurs between myoblasts to increase muscle mass. The additional nuclei acquired during fusion move apart from one another, positioning themselves with regular spacing throughout the length of the developing myotube. Finally, innervation and formation of the individual contractile muscle units are needed to allow transmission of neural inputs and movement. Collectively, these cellular processes lead to the formation of mature myofibers that support muscle function. Here, we add powerful support to a contribution of CF2 in the final myofibril size control. When CF2 was down or up regulated in the embryos, there were important changes in muscle size (Fig 5D–5F). Furthermore, we have observed absence of fibers in CF2 KD embryos (green arrows in Fig 5) while in CF2 OE embryos some fibers are duplicated (red arrow in Fig 5). In this context, we also noticed that, despite being smaller or larger fibers, their shape and anchoring points were not altered. Still more significant, in parallel to the size changes, we observed variations, reduction or increase in fiber nuclei number. A careful count of the nuclei present in the DA1 muscles of CF2 KD or OE embryos has allowed us to confirm and quantify those variations (Table 3 and Fig 6). Along with our conclusions, previous reported data obtained in adult flight muscles also suggested a contribution of CF2 in the control of the final size of indirect flight muscles [27].

In the context of previous data, it might be possible to discuss that variations in Mef2 expression, rather than in CF2, were the responsible for the described changes in nuclei number. However, data from others and us argue against this conclusion. Thus, while Mef2 loss of function completely blocks myoblast fusion and the expression of muscle differentiation program [10–13], a moderate over-expression does not cause an increase in nuclei number, but just some weak defects in muscle patterning together with the appearance of myosin expressing un-fussed myoblasts [42,43]. Interestingly, this last phenotype is somehow strikingly reminiscent of the very large accumulation of myosin expressing un-fussed myoblasts observed in embryos strongly overexpressing CF2 (Fig 3). To better support CF2 involvement in controlling muscle nuclei number we have used just one copy of Mef2>Gal4 driver to achieve a moderate Mef2 overexpression. DA1 muscles from stage 16 Mef2 OE embryos show the very same number of nuclei as control animals (Fig 6I/6J & Table 3), confirming the involvement of CF2 in the regulation of the number of nuclei present in each muscle fiber.

## Conclusions

Our demonstrations reveal two additional functions for CF2 not yet reported. First, CF2, is involved in the Mef2 transcriptional regulation. Second, CF2 acts at two closely related levels: contributing to the control of fiber size and to the number of nuclei that every fiber will have during embryo muscle differentiation.

## Supporting information

S1 Fig twi-Gal4 driven CF2 KD and OE phenocopies Mef2-Gal4 driven phenotypes. Lateral views of stage 17 embryos stained with anti-CF2 antibody are shown. **A.** *yw* embryo. **B.** twi/+>CF2i embryo. **C.** twi/twi>CF2i embryo. **D.** twi/+>CF2 embryo. **E.** twi/twi>CF2 embryo. Anterior to the left and posterior to the right. In panel B and D, + stands for *TM3Ser Act5C-GFP*. Pictures were collected using the same settings and images were equally processed. They correspond to maximum projections collected with maximum intensity.
(EPS)
